# Perfluoroalkyl substances in circum-Arctic*Rangifer*: caribou and reindeer

**DOI:** 10.1007/s11356-021-16729-7

**Published:** 2021-11-23

**Authors:** Anna Maria Roos, Mary Gamberg, Derek Muir, Anna Kärrman, Pernilla Carlsson, Christine Cuyler, Ylva Lind, Rossana Bossi, Frank Rigét

**Affiliations:** 1 Greenland Institute of Natural Resources, PO Box 570, 3900 Nuuk, Greenland; 2 Department of Environmental Research and Monitoring, Swedish Museum of Natural History, PO Box 50007, SE-10405 Stockholm, Sweden; 3 Gamberg Consulting, Box 11267, Yukon Y1A 6N5 Whitehorse, Canada; 4 Aquatic Contaminants Research Division, Environment and Climate Change Canada, 867 Lakeshore Road, Burlington, Ontario L7S 1A1 Canada; 5 MTM Research Centre, School of Science and Technology, Örebro University, SE-70182 Örebro, Sweden; 6 Norwegian Institute for Water Research (NIVA), Fram Centre, Hjalmar Johansens gate 14, 9007 Tromsø, Norway; 7 Department of Environmental Science, Aarhus University, Frederiksborgvej 399, 4000 Roskilde, Denmark; 8 Danish Centre for Environment and Energy, Department of Ecoscience, Arctic Research Centre, Aarhus University, Frederiksborgvej 399, 4000 Roskilde, Denmark

**Keywords:** Arctic terrestrial environment, Long-range transport, PFAS, PFOS, Canada, Greenland, Svalbard, Sweden

## Abstract

Livers of caribou and reindeer (*Rangifer tarandus*) from Canada (*n* = 146), Greenland (*n* = 30), Svalbard (*n* = 7), and Sweden (*n* = 60) were analyzed for concentrations of eight perfluoroalkyl carboxylic acids and four perfluoroalkane sulfonic acids. In Canadian caribou, PFNA (range < 0.01–7.4 ng/g wet weight, ww) and PFUnDA (<0.01–5.6 ng/g ww) dominated, whereas PFOS predominated in samples from South Greenland, Svalbard, and Sweden, although the highest concentrations were found in caribou from Southwest Greenland (up to 28 ng/g ww). We found the highest median concentrations of all PFAS except PFHxS in Akia-Maniitsoq caribou (Southwest Greenland, PFOS 7.2–19 ng/g ww, median 15 ng/g ww). The highest concentrations of ΣPFAS were also found in Akia-Maniitoq caribou (101 ng/g ww) followed by the nearby Kangerlussuaq caribou (45 ng/g ww), where the largest airport in Greenland is situated, along with a former military base. Decreasing trends in concentrations were seen for PFOS in the one Canadian and three Swedish populations. Furthermore, PFNA, PFDA, PFUnDA, PFDoDA, and PFTrDA showed decreasing trends in Canada’s Porcupine caribou between 2005 and 2016. In Sweden, PFHxS increased in the reindeer from Norrbotten between 2003 and 2011. The reindeer from Västerbotten had higher concentrations of PFNA and lower concentrations of PFHxS in 2010 compared to 2002. Finally, we observed higher concentrations in 2010 compared to 2002 (albeit statistically insignificant) for PFHxS in Jämtland, while PFNA, PFDA, PFUnDA, PFDoDA, and PFTrDA showed no difference at all.

**Supplementary Information:**

The online version contains supplementary material available at 10.1007/s11356-021-16729-7.

## Introduction

Per- and polyfluoroalkyl substances (PFAS) is the generic name for over 4500 chemicals including perfluoroalkyl acids (PFAAs) and their precursor compounds (OECD 2018), including perfluorinated carboxylic acids (PFCAs) and perfluorinated sulfonic acids (PFSAs), which are the most commonly analyzed PFAS. They are fluorinated, man-made chemicals with wide industrial and commercial applications (Kissa 2001). Being both oil and water repellent, PFAS are or have been used in a large number of different applications for over six decades, including in firefighting foams, grease-repellent food wrappers, textile stain and soil repellents, cleaning aids, processing aids in fluoropolymer manufacturing, ski wax, nonstick cookware, outdoor weather-repellent textiles, carpets and leather, etc. (Kotthoff et al. 2015; Prevedouros et al. 2006; Wang et al. 2013, 2015). They are tenaciously persistent in the environment, found in several abiotic and biotic remote Arctic samples (Muir et al. 2019), and released into the environment either directly or indirectly through their life cycle from industrial production to their usage in consumer products. Several episodes of direct release of PFOS into the environment because of firefighting activities have been documented (Li et al. 2018; Moody et al. 2002). Meanwhile, PFOA was used as a processing aid in the manufacturing of tetrafluoroethylene-based fluoropolymers. PFOA is also a degradation product of fluorotelomer precursors including fluorotelomer alcohols (FTOHs) which are used to make polyfluoro-based products.

The largest producer of PFOS and PFOS-based compounds, the 3M Company, voluntarily phased out their production of PFOS in 2001 after evidence of elevated PFOS concentrations in blood from employees as well as in wildlife (3M Phase-out plan for POSF-based products, 2000; Giesy and Kannan 2001), and the European Union restricted the use of PFOS in 2006 (EC 2006). From the mid-2000s, production has increased in China (Xie et al. 2013). In 2009, PFOS, its salts, and perfluorooctane sulfonyl fluoride (PFOS-F, which degrade to PFOS in the environment) were added under Annex B of the Stockholm Convention of Persistent Organic Pollutants (POPs) (http://www.pops.int/) and PFOA was later added to Annex A.

In terms of toxicity of PFAS, PFOS is the most investigated PFSA, while PFOA is the most investigated PFCA. The highest concentrations of PFOS and PFOA in humans and wildlife are usually found in the liver, kidney, and blood (Butt et al. 2010; Houde et al. 2011). PFOS has a negative effect on the immune system (Budtz-Jørgensen and Grandjean 2018; De Witt et al. 2012; Petersen et al. 2018). Furthermore, PFOS and PFOA have been shown to cause increased liver weight and hepatocytic hypertrophy (Kennedy et al. 2004; Seacat et al. 2003), abnormal behavior, weight loss, and serious damage in liver and lung tissue (Cui et al. 2008) as well as developmental neurotoxic effects (Johansson et al. 2008).

PFAS occurrence in humans and wildlife is global (Butt et al. 2010); they are found in the Antarctic environment (Schiavone et al. 2009) as well as the Arctic (Muir et al. 2019). Most wildlife studies have focused on the aquatic food chain, where the highest concentrations usually are found. Studies of PFAS in Arctic terrestrial animals are relatively few (Aas et al. 2014; Bossi et al. 2015; Butt et al. 2010; Larter et al. 2017; Martin et al. 2004; Muir et al. 2019; Müller et al. 2011; Routti et al. 2017).

Caribou and reindeer are the same species, *Rangifer tarandus*, but there are several subspecies. They are an important food resource for people throughout the Arctic and an intrinsic component of traditional culture and food for many northern peoples. PFOS and perfluorinated carboxylates (PFCA C_7_–C_11_) were measured in traditional foods collected in Nunavut, Canada, between 1997 and 1999. Highest concentrations of total PFAS were found in caribou liver and the authors concluded that caribou meat contributed 43–75% of daily PFAS dietary exposure in the area (Ostertag et al. 2009). With their circumpolar distribution, caribou and reindeer are a suitable species for investigations of spatial trends across the Arctic. The main transport route for PFAS, which are directly released into aquatic systems, are ocean currents, whereas volatile precursors of PFSA and PFCAs undergo long-range atmospheric transport and degrade to PFSA and PFCAs in the atmosphere and cryosphere (see Muir et al. 2019 and references therein). Furthermore, sea spray aerosols have been shown to be an important mechanism for global transport of PFAS from the marine to coastal terrestrial environments (Johansson et al. 2019).

Arctic communities themselves can be a local source of PFAS in their environment. In Svalbard, firefighting training sites and landfill locations were identified as major PFAS sources (Skaar et al. 2019). Also, at the community of Resolute Bay in the Canadian Arctic, elevated concentrations of PFOS were found in soils near the local airport and former military base, and these were attributed to presence in firefighting foams (Cabrerizo et al. 2018). Similarly, and relative to an area nearby, elevated concentrations of mainly PFOS occurred in the marine environment surrounding a military site in Norway (Landberg et al. 2019). These examples demonstrate the importance of local sources.

The purpose of this study was to illuminate similarities and differences in PFAS levels and patterns in livers from *R. tarandus* spp. across the Arctic and, where data allowed, also to analyze temporal trends. Information pertaining to PFAS in Russian *Rangifer* is to our knowledge unavailable and therefore we have focused on North American, Greenland, and European arctic. We analyzed caribou samples from seven areas in northwestern Canada (*n* = 146) and two areas in southwest Greenland (*n* = 20). We also analyzed reindeer from Isortoq (south Greenland, *n* = 10) and Svalbard (*n* = 7) and three areas in central and northern Sweden (*n* = 60). We focused on eight PFCA and four PFSA and the precursor PFOSA.

## Materials and methods

### Sample collection

**Fig. 1:**
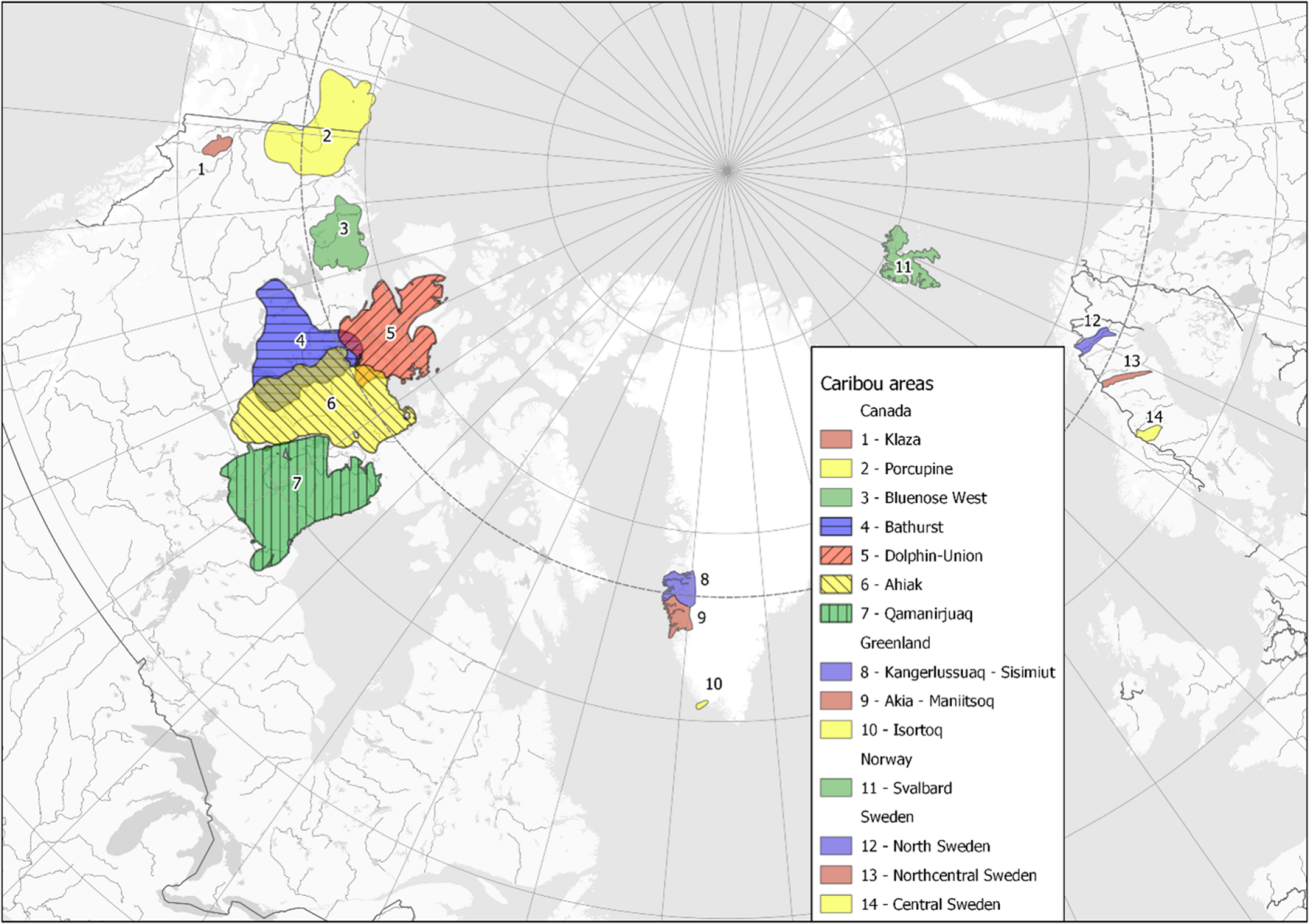
Map indicating the different sampling areas. Norrbotten = #12 North Sweden, Västerbotten = #13 north-central Sweden, and Jämtland = #14 central Sweden

Altogether, 243 liver samples from caribou and reindeer were analyzed. Information on sampling years, size, and local areas can be found in Table 1 and Fig. 1. PFAS are associated with protein-rich tissues and not lipids. Hence, all concentrations are given based on wet weight (ww) of the samples. Since different laboratories conducted the analyses, the LOD (limit of detection) differ slightly between the studies and we have taken that into account when comparing results.

**Table 1:** Collection year, sex, and age (range and mean) of the specimens analyzed within this study

Country	Area	Year	*n*	Males	Females	Unknown sex	Age (years) range and mean	Unknown age
Canada	Klaza	2002–2005	4	3		1	4, 5	2
	Porcupine	2005	5	5			2–4 (3.2)	
		2006	10	8	2		3–6 (4.9)	
		2007	10	9	1		3–7 (5)	4
		2008	10	10			3–10 (6.7)	
		2011	10	10			3–7 (5)	
		2015	10	10			3–7 (5.4)	
		2016	10	10			5–10 (7.1)	
	Bluenose West	2015	10	9	1			10
	Bathurst	2008	7		7		4–11 (7)	
	Dolphin & Union	2015	10	10			3–10 (6.4)	
	Ahiak	2016–2017	10	3	7		0.5–12 (3.2)	
	Qamanirjuaq	2008	10	6	4		5–11 7.7)	
		2011	10	10			0.5–10 (4.7)	
		2015	10	4	6		2–11 (6.7)	
		2016	10	5	5		3–12 (7.3)	
Greenland	Akia-Maniitsoq	2008	10	0	10		1.8–9.8 (5)	
	Kangerlussuaq-Sisimiut	2009	10	0	10		2.8–11.8 (8)	
	Isortoq	2012	10			10	3–5	2
Norway	Svalbard	2010	7	2	3	2	0.5–2.5 (1.2)	
Sweden	Norrbotten	2003	10	10			3.3	
		2011	10	10			3.3	
	Västerbotten	2002	10	10			3.3	
		2010	10	10			3.3	
	Jämtland	2002	10	10			3.3	
		2010	10	10			3.3

Caribou from Canada and reindeer from Sweden and Greenland were collected within their respective national programs: Canada’s Northern Contaminants Program, Sweden’s National Monitoring Program (administered by Swedish Museum of Natural History), and the Danish Cooperation for Environment in the Arctic (DANCEA). Samples from Svalbard were collected from local hunters.

Livers from all areas were sampled at harvest and stored at −25 °C until a subsample was collected for analysis. Chemical analyses on the liver samples were conducted at laboratories specific to the country where the samples were collected, i.e., Canada (including southwest Greenland), Denmark (Isortoq, south Greenland samples), Norway, and Sweden. Altogether, 8 PFCA (PFOA, PFNA, PFDA, PFUnDA, PFDoDA, PFTrDA, PFTeDA, and PFHpA) and 4 PFSA (PFHxS, PFOS, PFDS, and the precursor FOSA) were analyzed within this study. For full names of the acronyms, see Supporting information, Table S1. Portions of the data have been published previously (Bossi et al. 2015; Müller et al. 2011).

### Analytical methods

The methods used have been previously published (Müller et al. 2011) and are similar for all samples except those from Isortoq (South Greenland), where an ion-pairing method was used for extraction (Bossi et al. 2015). In general, the liver samples were thawed, and a representative subsample was prepared for acetonitrile-based extractions followed by clean-up with graphite carbon solid-phase cartridges (Canada) or dispersive graphitized carbon (ENVI-Carb) and glacial acetic acid (Sweden and Norway). All laboratories used isotopically labeled (mainly ^13^C) internal standards (IS) covering a suit of C_6–14_ PFSA, PFCA, and PFOSA. For the exact IS used in each laboratory, see Lescord et al. (2015); for Canada and southwest Greenland, Carlsson et al. (2014), and Herzke et al. (2009) for Norway; and Eriksson et al. (2016) for Sweden. All laboratories used high-performance liquid chromatography (HPLC) coupled to tandem mass spectrometry (MS/MS) systems with negative electrospray ionization (ESI) for analyses, although there were minor differences in the setup, columns, and brands between the laboratories (see SI Table S2 for a summary of the methods used).

### Quantification and quality assurance

The quantification programs and methods, including definitions of, e.g., limits of detection, varied slightly between each sample batch, but are thoroughly described in Carlsson et al. (2014) for Norway, Müller et al. (2011) for Canada and Southwest Greenland, and Bossi et al. (2015) for Isortoq, South Greenland. Samples were quantified with a five (Sweden) or six (Canada) point calibration curve and isotopic dilution method. All laboratories used their internal quality assurance guidelines, as described, and referred below. The Canadian laboratory analyzed certified reference materials (CRM), NIST 1946 Lake Superior Fish Tissue (Reiner et al. 2012), with each batch of 10 samples and the recoveries from certified values of PFOS averaged 113 ± 32% (*n* = 18). The Norwegian laboratory also analyzed a CRM (PFAS ILS 2011; fish tissue). The CRMs analyzed were within an acceptable range of stated values (±20%). The Swedish laboratory included an in-house quality control sample (fish muscle) in each batch to assess the reproducibility and accuracy (see SI Table 2).

**Table 2:**
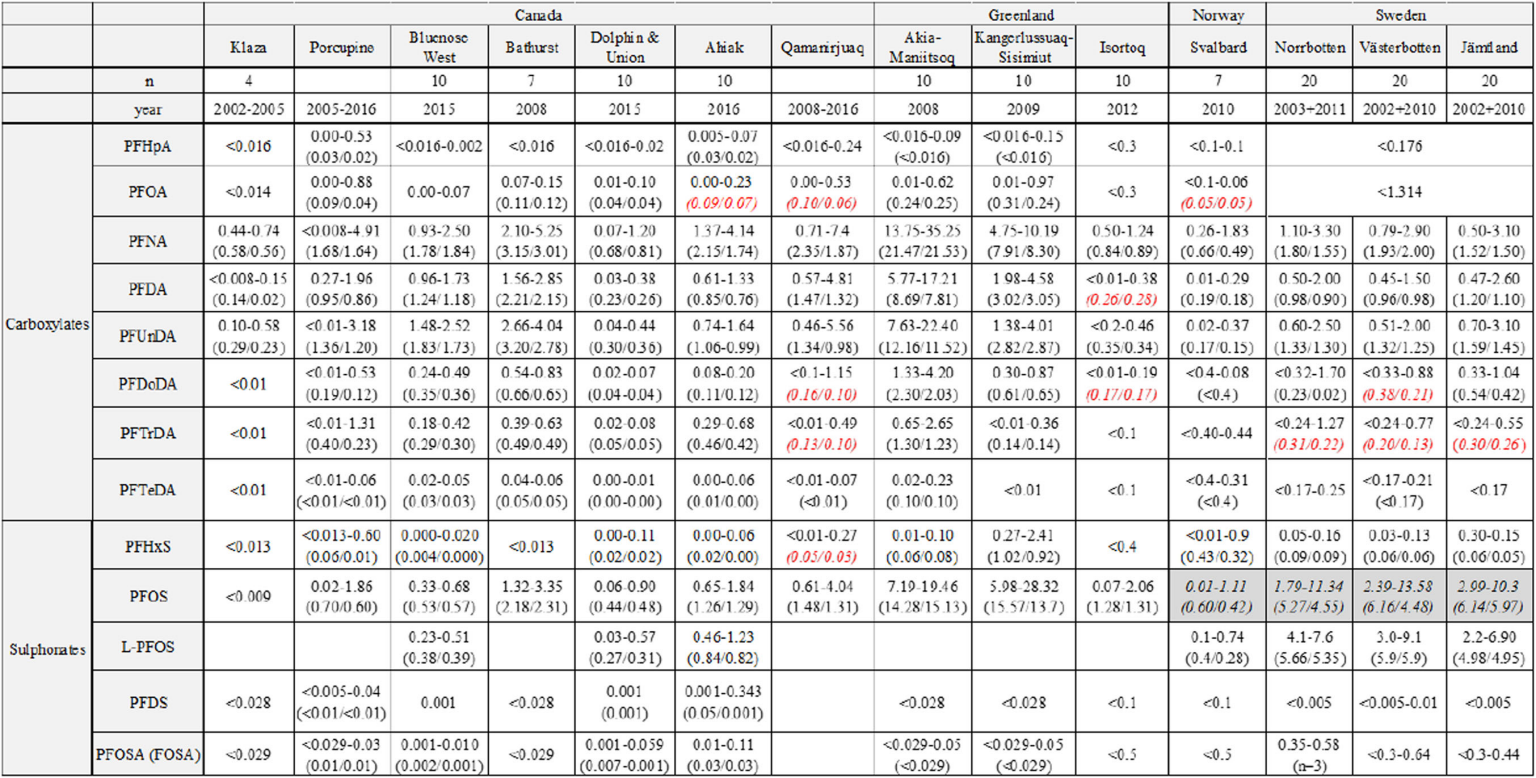
Range (mean/median) of PFAS (ng/g ww) in caribou and reindeer populations. Values in bold italics are calculated mean and median values using Regression on Order Statistics (ROS). Italicized in gray box are estimated PFOS from L-PFOS

The limit of detection (LOD) was in general defined as a signal-to-noise ratio (S/N) of 3:1 and in the case of detection of target compounds in the blanks, the mean concentration in blank samples with addition of three times the standard deviation was considered LOD. Values of LOD for all compounds and laboratories are provided in Supporting information, Table 1.

All laboratories except for Örebro University in Sweden and NILU in Norway reported total-PFOS(PFOS) whereas Örebro University and NILU reported linear-PFOS (L-PFOS). For the Canadian liver data from 2015, both PFOS and L-PFOS were available (*n* = 79). For these data, the amount of L-PFOS was ca 64 ± 6% (mean ± SD) of total PFOS. This mean ratio was used to convert the Swedish and Norwegian L-PFOS data to allow comparisons between geographical areas.

### Statistical methods

We used regression on order statistics (ROS)(Helsel 2012) to estimate means and medians for each population with less than 80% of the values < LOD. No estimation of means and medians was done if more than 80% of the values were < LOD, following Helsel’s recommendation that the number of censored data should not be above 80% when having sample size <50 (Helsel 2012). Any values below the LOD (non-detections) were replaced by LOD/√2 in cases with maximum of 20% of the samples being less than LOD (Loftis et al. 1989). Prior to statistical analysis, concentration values were log-transformed to approach the assumptions of normal distribution and variance homogeneity. *P*-values below 0.05 were considered statistically significant and biologically relevant, while *P*-values between 0.05 and 0.1, albeit insignificant, do not necessarily imply that a biologically relevant tendency was not present.

Analysis of variance (ANOVA) was performed to test for differences in PFAS compounds between the populations. The tests of the explanatory variables were based on Type III Sum of Squares, where each term is evaluated after all factors have been accounted for (i.e., partial, not sequential). Tukey’s post hoc tests for comparisons of means were used to detect significant pairwise differences among predictor variables.

Temporal trends were investigated using those populations that were sampled in multiple years: Porcupine and Qamanirjuaq caribou in Canada (between 2005–2016 and 2008–2016 respectively) and Swedish reindeer (Norrbotten, Västerbotten, and Jämtland, sampled in 2002/2003 and in 2010/2011, see Table 3). Regarding the Porcupine and Qamanirjuaq caribou, temporal trend was analyzed using a robust method, in which annual median concentrations were used as index values. The median was chosen to minimize the influence of outliers and values below detection limits. The method tests for the presence of a log-linear trend and/ornon-linear trend by separating the total variance over time into a log-linear component and a non-linear component (Nicholson et al. 1998). The log-linear trend was tested by log-linear regression. A 3-point running smoother was applied to describe the nonlinear trend component and tested by means of ANOVA. Mood’s median test was applied for each of the Swedish reindeer populations (Norrbotten, Västerbotten and Jämtland) with two sample occasions to test for differences between sampling years. All statistical analyses were performed using R (R Core Team 2019).

**Table 3:**
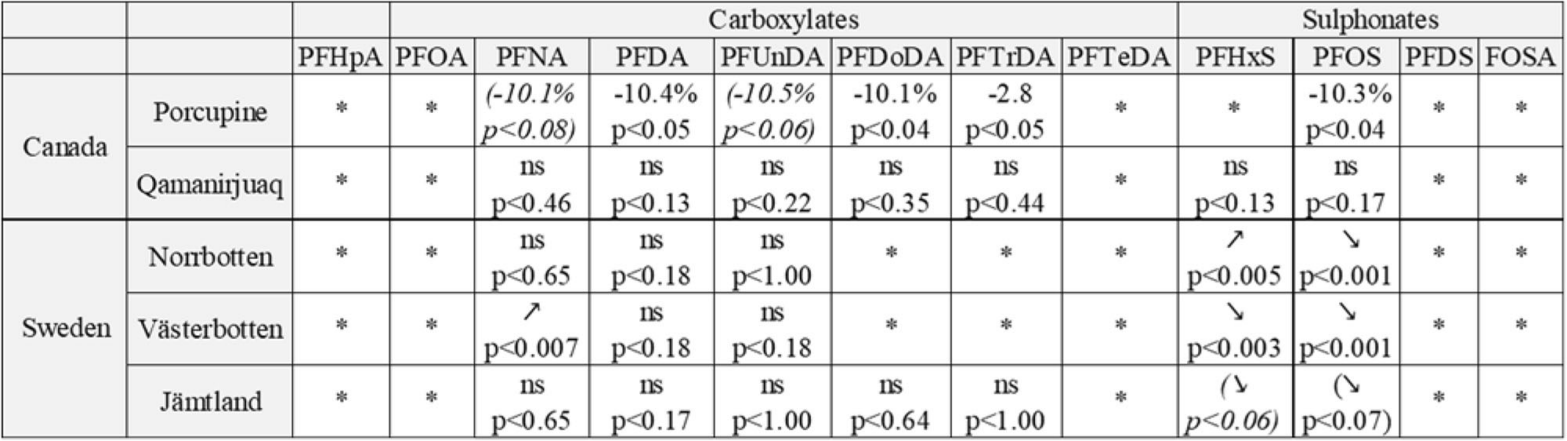
Trend analyses for two Canadian and three Swedish populations. The Porcupine population was sampled on several occasions, so it was possible to calculate a yearly change in %. Values in parenthesis are trends, with *p* values between 0.05 and 0.1. * = too many values under MDL to allow statistical analyses

## Results

**Fig. 2:**
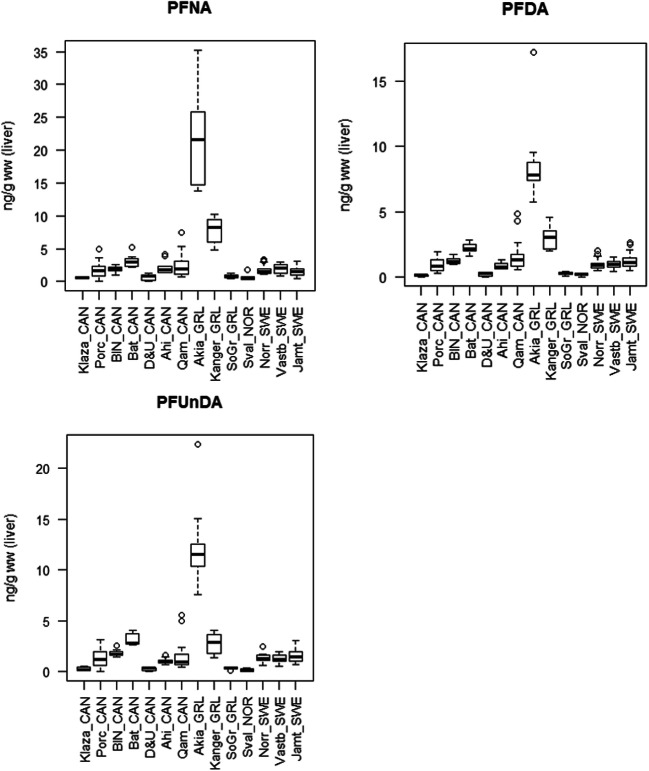

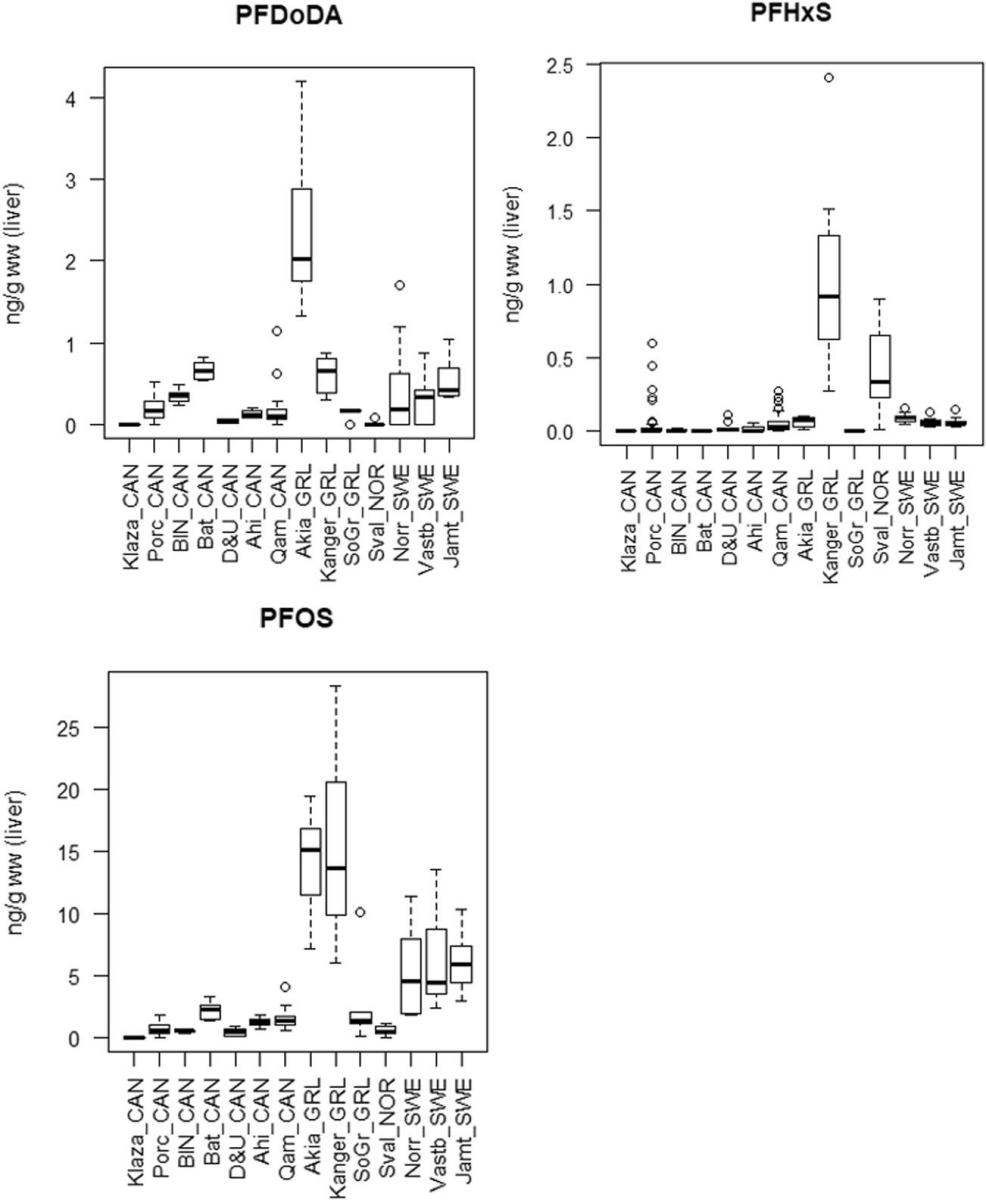
Box plots showing concentrations of PFNA, PFDA, PFUnDA, PFDoDA, PFHxS, and PFOS in fourteen caribou and reindeer populations. The horizontal thick lines show median values. The hinges represent the first and third quantile. The notches represent roughly 95% confidence interval for the median values. Outliers are shown in circles. From left to right: seven populations in Canada (CAN)—Klaza (*n* = 4), Porcupine (Porc, *n* = 65), Bluenose West (BN, *n* = 10), Bathurst (Bat, *n* = 7), Dolphin & Union (D&U, *n* = 10), Ahiak (Ahi, *n* = 10), Qamanirjuaq (Qam, *n* = 40); three populations in Greenland (GRL)—Akia-Maniitsoq (Akia, *n* = 10), Kangerlussuaq-Sisimiut (Kanger, *n* = 10), and Isortoq (SoGr, *n* = 10). Svalbard, Norway (Sval_NOR, *n* = 7); and finally three populations in Sweden (SWE)—Norrbotten (Norr, *n* = 20), Västerbotten (Vastb, *n* = 20), and Jämtland (Jamt, *n* = 20)

**Fig. 3:**
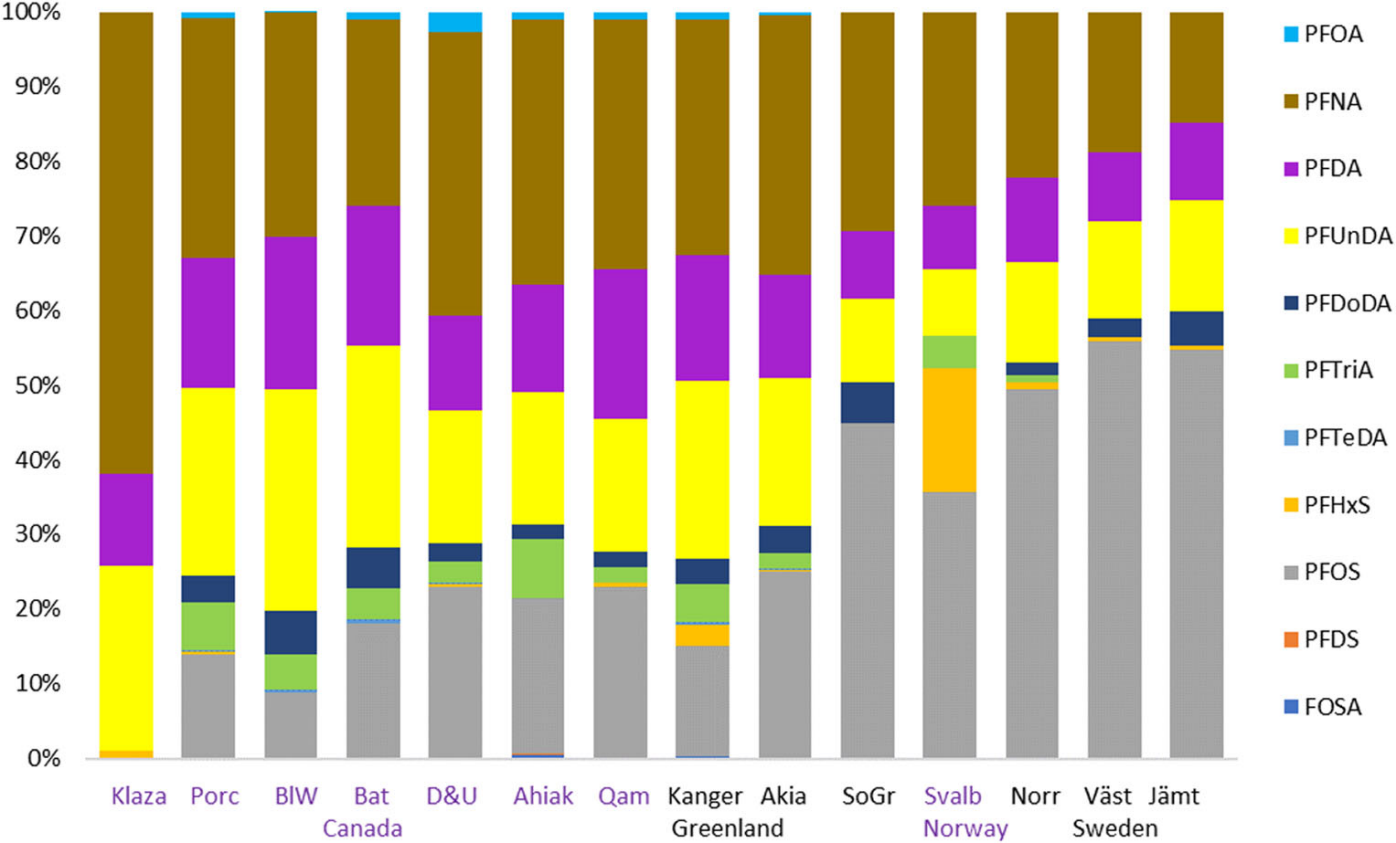
The pattern of PFAS compounds in liver tissue from caribou/reindeer indicating PFCA (i.e., PFNA, PFDA, and PDUnDA) dominated in the Canadian Arctic and western Greenland while PFSA (i.e., PFOS) dominated in Svalbard, Sweden, and Isortoq

**Fig. 4:**
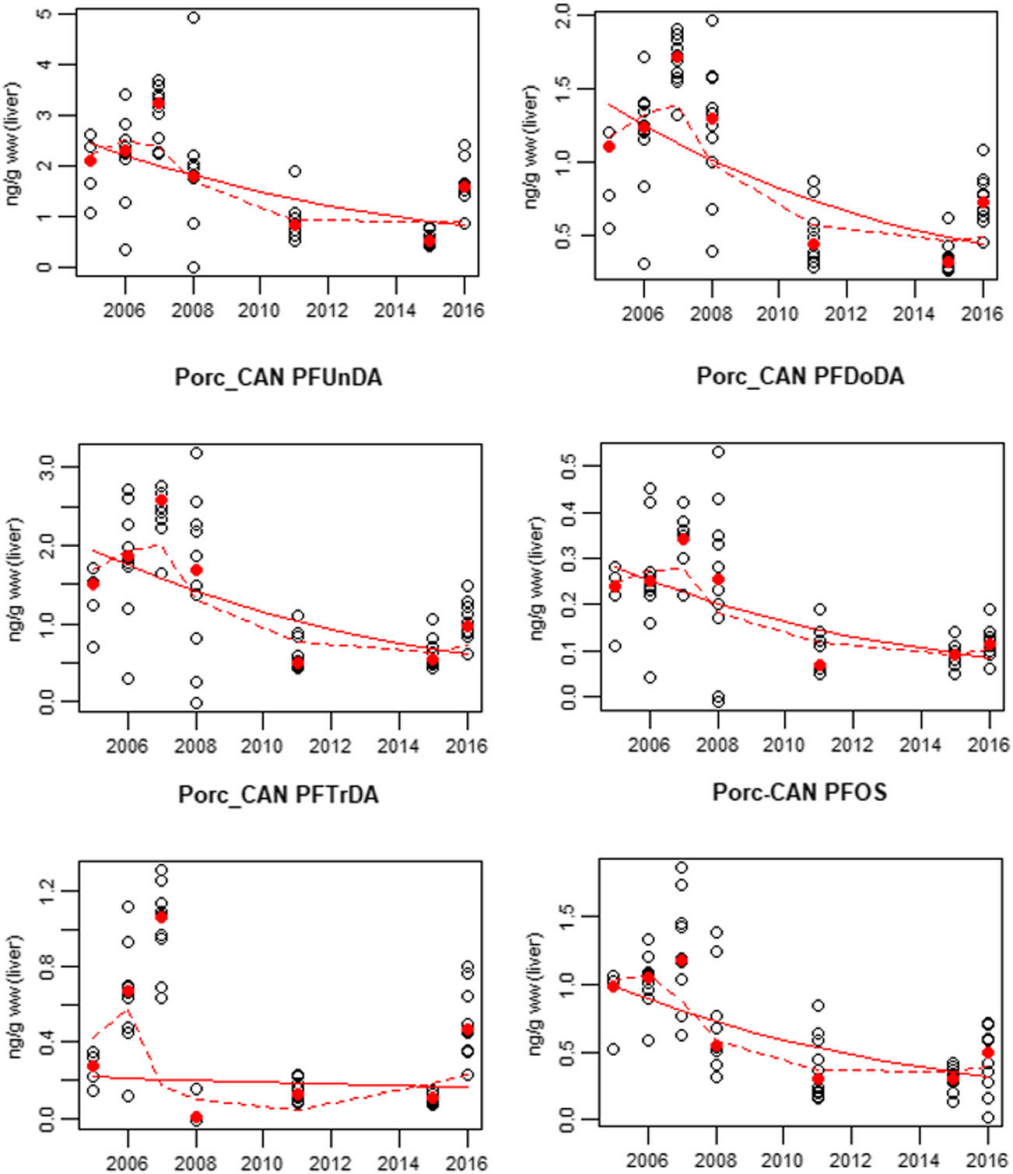
Time trends for PFDA, PFDoDA, PFTrDA, and PFOS in Porcupine caribou, NW, Canada, show significant yearly decreases of ca 10% except for PFTrDA (only 3% yearly decrease). PFNA and PFUnDA show decreasing trends, however not statistically significant. The solid red line represents a log linear regression and the dashed red line a 3-year smoother. Open circles represent individuals and filled circles are the annual median OBS The titles of the first two graphs are missing! Should read "Porc_CAN PFNA and Porc_CAN PFDA

Range, mean, and median values for all populations are shown in Table 2. Figure 2 shows boxplots of all populations while Fig. 3 shows the pattern of compounds within each population, Fig. 4 shows time trends for Porcupine caribou, and Fig. S1 in Supporting Information shows time trends for Qamanirjuaq caribou.

### Age and sex

The caribou and reindeer livers analyzed in this study were collected under varying circumstances. In Sweden, 3-year-old bulls were collected during the annual September slaughter of semi-domestic reindeer. When hunting wild animals (Svalbard, Greenland, Canada), it was not possible to attain the same level of standardized collection regarding season, age, and sex. The mean age of all caribou sampled was 5 years old (range 1–12). Robust analyses of sex with PFAS were not possible. As shown in Table 1, the majority of the collected samples were male. No pronounced relationship with age was observed; hence, those variables were not included in the statistical analyses.

### Concentrations of PFAS

Relatively low and similar concentrations of most PFAS were observed in Canada and Isortoq (South Greenland), and for most PFAS also Kangerlussuaq-Sisimiut (Kanger), Svalbard, and Sweden compared to Akia-Maniitsoq(Akia) caribou having elevated concentrations of all PFAS except for PFHxS (see Fig. 2). Elevated concentrations of PFOS were seen in both the Akia and Kanger populations (up to 28 ng/g ww) and in the Swedish populations (up to 14 ng/g ww). All other populations had concentrations below or well below 4 ng/g ww. In contrast, PFHxS showed a different pattern, with Kanger caribou (2.4 ng/g ww) and some individual caribou from Svalbard (up to 0.9 ng/g ww) having the highest concentrations. Other samples were usually below 0.2 ng/g. Concentrations of PFNA were elevated in both populations from Southwest Greenland, in the Akia population up to 35 ng/g (median 21.5 ng/g) and the Kanger population up to 10 ng/g ww (median 8.3 ng/g). In all other populations, the concentrations were most often below 5 ng/g ww (medians between 0.6 and 3.0 ng/g ww).

Furthermore, concentrations of PFDA and PFUnDA were under 5 ng/g in all specimens with the exception of Akia caribou (PFDA up to 17.2 and median 7.8 ng/g and PFUnDA up to 22.4 ng/g and median 11.5 ng/g). As for PFDoDA, all but Akia specimens had concentrations 1.2 ng/g or below, with one exception (one reindeer from Norrbotten, Sweden, had 1.7 ng/g ww). In Akia caribou, the concentrations of PFDoDA were between 1.3 and 4.2 ng/g (median 2.0 ng/g) (Fig. 2).

For central Arctic Canada, a weak spatial trend can be seen with slightly increasing median concentrations of most PFAS from Klaza caribou in the west to Bathurst caribou further east. Also, the Dolphin & Union caribou in the far north generally had very low concentrations, while further east and south, higher concentrations were observed in Ahiak and then Qamanirjuaq caribou (Table 2 and Fig. 2). Within Canada, Bathurst caribou had the highest concentrations of PFOS, PFNA, PFDA, PFUnDA, and PFPoDA.

### Relative distribution of PFAS compounds

The pattern of PFAS compounds from caribou/reindeer shifted from PFCA dominated in the Canadian Arctic and western Greenland to PFSA dominated in Svalbard, Sweden, and Isortoq, South Greenland (Fig. 3). The long-chain PFCAs (C_9–11_; PFNA, PFDA, and PFUnDA) were the most dominant compounds in caribou from Canada, while PFOS dominated samples from Isortoq, Svalbard, and Sweden, followed by PFNA.

### Time trends

Temporal trends in several PFAS were evident in caribou from one of the two Canadian populations that was sampled multiple years, as well as the three populations in Sweden (Table 3, Fig. 4 and Fig. S1 in Supporting Information). In Porcupine caribou, concentrations of PFNA, PFDA, PFDoDA, PFUnDA, PFTrDA, and PFOS decreased significantly at a rate of approximately 3–10% annually between 2005 and 2016. PFTrDA first increased and then decreased during the study period, ending at approximately the same level as in the beginning of the time series. An exponential description of the temporal trends was appropriate since non-linear trends components were not significant. As for Qamanirjuaq caribou, no significant trends were seen, likely due to low statistical power with data from only 4 years. Concentrations of PFTrDA increased from mostly below the LOD in 2008 (<0.01) to slightly above the LOD in recent years, including in Qamanirjuaq, indicating an increase in concentrations (median concentration in 2016 was 0.10 ng/g ww, Fig. S1).

Reindeer from the three Swedish populations were sampled on two occasions each, in 2002/2003 and in 2010/2011. Lower concentrations in recent years were observed for PFOS in the two northernmost populations (Norrbotten and Västerbotten, *p* < 0.001), and a decreasing trend was seen in the southernmost population (Jämtland) (*p* < 0.07). PFHxS increased in concentrations in the northern population (Norrbotten, *p* < 0.005) and decreased in Västerbotten (*p* < 0.003) and Jämtland (*p* < 0.06). PFNA concentrations increased in Västerbotten reindeer (*p* < 0.007), although this trend was not observed for the other two populations. No changes in concentrations of PFDA and PFUnDA were observed for any of the three populations. Concentrations of PFDoDA and PFTrDA had too many values below LOD to be able to analyze statistically in the two northern populations (Norrbotten and Västerbotten), and in reindeer from Jämtland, central Sweden, the concentrations were stable.

## Discussion

### Age and sex

No pronounced relationship was seen between PFAS concentrations, sex, and age in this study. This agrees with several other studies of other mammals (Persson et al. 2013, Roos et al. 2013,Smithwick et al. 2005, Butt et al. 2008, Routti et al. 2016, Shaw et al. 2009, Houde et al. 2006, Kowalczyk et al. 2018).

### Concentrations of PFAS

PFOA was found in low concentrations in all study samples. Indeed, all caribou/reindeer had similar or lower concentrations of PFOA than roe deer (*Capreolus capreolus*) from Germany (Falk et al. 2012) and beavers (*Castor fiber*) from Poland (et al. 2007). Chamois (*Rupicapra rupicapra*) from Austria and red fox (*Vulpes vulpes*) from Germany also had very low concentrations of PFOA, most often below the LOD (Riebe et al. 2016).

Caribou from the two areas in Southwest Greenland, specifically Akia but also Kanger, stand out compared to all other populations in this study by having the highest concentrations of PFOS, PFNA, PFDA, PFUnDA, and PFDoDA. The home range of the Kanger caribou is close to an international airport and a former military base (an American operated base between 1941 and 1992) and the Akia caribou live close to the capital Nuuk. In contrast, the other sampled populations in this study live in relatively remote areas. The proximity to likely local point sources may be an important explanation of the general high concentrations of PFAS (Landberg et al. 2019*;* Slinde and Høisæter 2017; Stock et al. 2007; Skaar et al. 2019). However, there are marked differences in the pattern of PFAS compounds between the two neighboring Greenland populations. While the PFOA and PFOS concentrations were similar, the Akia caribou had considerably higher concentrations of PFNA, PFDA, PFUnDA, and PFDoDA compared to caribou in Kanger. In contrast, Kanger caribou had higher concentrations of PFHxS. Further, PFHxS showed a different pattern compared to other PFAS. The concentrations were low and similar in Akia caribou, Canada, and Sweden, but levels were somewhat elevated in some Svalbard reindeer and highest in Kanger caribou. The LOD was too high (0.4 ng/g) in samples from reindeer from Isortoq, South Greenland, to allow a comparison. Kanger caribou stand out as having the highest concentrations of PFHxS of any population sampled and much higher than even their neighboring Akia caribou. This may be related to activities at the former American military base, as well as usage of PFHxS during operation of the Kanger International Airport. Besides the proximity to possible local sources, the two areas of Southwest Greenland differ markedly in climate and vegetation types. The Akia caribou live in a wet maritime climate with lots of lichens. The Kanger caribou live in an inland habitat which is a dry continental desert steppe, dominated by dwarf shrub heath and grasslands with almost no lichens (Gamberg et al. 2016). The resulting differences in diets may be a part of the explanation of the differences in contaminant concentrations between these populations. Müller et al. (2011) studied PFAS in a terrestrial food chain in two remote areas of northern Canada and found that the pattern of PFAS differed greatly between vascular plants and lichen. The highest average ΣPFCA concentrations in the two study areas were found in lichen (*Cladonia mitis/rangiferina* and *Flavocetraria nivalis/cucullata*) and Arctic willow (*Salix pulchra*).

Aastrup et al. (2000) observed differences in heavy metal concentrations in caribou from Akia and Kanger and also related that to differences in occurrence of vegetation types and subsequent dietary differences. An additional source for the Akia could be sea spray aerosols which impact grazing areas near open seas (Johansson et al. 2019), which are improbable for inland Kanger. A study analyzing Arctic air samples from Alert (Canada, 2006–2014), Zeppelin (Svalbard, Norway, 2006–2014), and Andøya (Norway, 2010–2014) showed that PFCA were 3 to 30 times higher in the Norwegian samples compared to Alert (Wong et al. 2018). This was explained by the fact that the two Norwegian sites are located closer to the open ocean and more exposed to sea spray aerosol. In the present study, concentrations were generally higher in the European sites compared to Canada, which could be explained by both sea spray aerosol (Akia and Svalbard) and closer proximity to contaminated areas (i.e.*,* human activities) in Sweden and Southwest Greenland.

The median concentrations of PFOS in Akia and Kanger (15 and 14 ng/g) are lower compared to levels in marine mammals in Greenland and Svalbard (Aas et al. 2014; Boisvert et al. 2019; Rigét et al. 2013; Routti et al. 2017; Routti et al. 2019) as well as mammals and aquatic birds from Canada (Martin et al. 2004; Braune et al. 2014). The concentrations of PFOS in this study were highest in Southwest Greenland and Sweden. A somewhat similar circumpolar trend was seen for polar bears, where the lowest concentrations were found in Alaska, higher concentrations further east in Hudson Bay and East Greenland, and the highest in Barents Sea (Routti et al. 2019). However, caribou from Southwest Greenland had much higher concentrations than what was found in livers from terrestrial herbivores in Europe, such as roe deer from Germany sampled the same year (ca 2 ng/g, Falk et al. 2012), and chamois from Austria (2.4 ng/g; Riebe et al. 2016) as well as beaver from Poland (2.4 ng/g; Falandysz et al. 2007), moose (*Alces alces*) from Norway (range 0.18–0.39, mean 0.27 ng/g ww; Hanssen et al. 2019), and northern Canada (Larter et al. 2017). The median concentration of PFHxS in Kanger caribou (1 ng/g) and Svalbard reindeer (0.3 ng/g) was higher than median concentrations in German roe deer (<0.5 ng/g, Falk et al. 2012), and in beavers from Poland (<0.001 ng/g; Falandysz et al. 2007). The reason for elevated levels of PFHxS in Kanger caribou and some of those from Svalbard is not known, but contamination from local sources, such as firefighting exercises, cannot be ruled out. European chamois and red fox had lower concentrations of PFHxS, most often below the LOD although they are closer to more urban areas (Riebe et al. 2016). The level of PFNA in Akia caribou (mean 21 ng/g) was lower than from polar bears in Greenland but similar to what was found for ringed seals in East Greenland (2011–2012) (Boisvert et al. 2019) and similar to mink and arctic fox (*Vulpes lagopus*) in Canada (means 16 and 22 ng/g ww, Martin et al. 2004) but higher compared with seals and birds in Canada (means <0.05–5.9 ng/g ww, Martin et al. 2004). In addition, concentrations of PFNA in Akia was much higher compared to European values for roe deer (1.2 ng/g, Falk et al. 2012), chamois, and red fox (1.9 and 1.3 ng/g respectively, Riebe et al. 2016) and beavers (0.12 ng/g, Falandysz et al. 2007). It is not surprising that caribou have lower concentrations compared to animals such as seals and polar bears who are feeding on a higher trophic level, but it is unexpected that they have higher concentration compared to terrestrial mammals specifically herbivores in Europe.

### Relative distribution of PFAS compounds

PFNA was the dominant PFAS in caribou from Canada and Southwest Greenland together with PFUnDA and PFDA and found in elevated concentrations in specially Akia. In Canada and Southwest Greenland, PFNA and PFUnDA accounted for usually more than 50% of ΣPFAS, and together with PFDA more than 70%. A similar pattern was seen in moose from the southern northwest territories of Canada, where PFNA was the predominant PFCA (Larter et al. 2017).

In contrast to the present study’s Canadian caribou livers, the most dominant PFCA in five species of birds (eggs) from Arctic Canada were PFUnA and PFTrA, except for black guillemot (*Cepphus grylle*), where PFDA contributed almost equally with PFTrA to the PFCA profile (Braune and Letcher 2013). In the present study, PFTrA did not contribute significantly to any sample. Previous data has shown an even-odd pattern of PFCA in mammals in remote locations, where by the odd > even for sequential pairs of PFCA as attributed to long range transport of volatile precursors (Martin et al. 2004).

In contrast to caribou from Canada and Southwest Greenland, PFOS was the dominating PFAS in Isortoq (South Greenland), Svalbard, and Sweden. This is more in line with many other studies on PFAS in other wildlife (e.g., Butt et al. 2010; Aas et al. 2014; Falk et al. 2012; Boisvert et al. 2019). However, the situation is changing. Global wildlife sampling over a decade ago uniformly indicated a predominance of PFOS in PFAS congener profiles. However, more recent studies show that long-chain PFCAs are increasing in concentration while PFOS is declining. Therefore, the gap in concentration between long-chainPFCA and PFOS is becoming smaller. For example, Villanger et al. (2020) showed that Svalbard beluga have 14 ng/ml total PFCA and 25 ng/ml PFSA in 2013–14 whereas 15 years earlier the concentrations were 9 ng/ml PFCA and 43 ng/ml PFSA. This difference can be attributed to greater regulatory controls on PFOS in the early 2000s and delayed action on PFCA and their precursors.

### Time trends

Concentrations of PFDA, PFDoDA, PFTrDA, and PFOS decreased significantly between 2005 and 2016 in Porcupine caribou (*p* < 0.05) and a decreasing, not significant, trend was seen for PFNA (*p* < 0.08) and PFUnDA (*p* < 0.06). No statistically significant trends were seen in Qamanirjuaq caribou between 2008 and 2016, probably due to low power in the statistical analyses (see Fig. S1 in supporting information). Similarly, concentrations of PFOS in the three Swedish populations were lower in 2010/2011 compared to the early 2000s. Concentrations of PFHxS in reindeer from Norrbotten were higher in 2011 compared to those in 2003 in contrast to the two more southern populations, where the concentrations were lower in 2010 compared to 2002. In Västerbotten, concentrations of PFNA were higher in 2010 compared to those in 2002. For other PFCA, no statistical change in concentrations was observed in the Swedish reindeer. This may be owing to the few years available for temporal trends. Meanwhile, recent studies show that PFAS concentrations in Swedish otters are either still increasing or stable (Roos and Benskin 2016). Other investigations have reported decreasing concentrations of PFOS and other PFAS (Muir et al. 2019; Rigét et al. 2013), as a consequence of the phasing out of PFOS production in USA around 2002 (3M Phase-out plan for POSF-based products, 2000; Giesy and Kannan 2001) as well as the restricted use of PFOS in Europe and Canada shortly thereafter (EC 2006) and the phase out of PFOA by fluorochemical manufacturers in the USA and Europe under the US EPA Stewardship program (USEPA 2006). In East Greenland, annual average concentrations of PFOS in polar bear liver peaked in 2006, at 2966 ng/g ww. The same trend was found for ringed seal liver from both West and East Greenland, where concentrations peaked in 2006 (with a mean of 352 ng/g ww in East Greenland and 397 ng/g in West Greenland, Rigét et al. 2013). A similar pattern was seen in Porcupine caribou in the present study, where concentrations peaked in 2007. Concentrations of PFOS in bird eggs from Prince Leopold Island in Arctic Canada 1975–2012 peaked in 2008 (Braune et al. 2014). Declining trends of PFOS were also observed in ringed seals from the Canadian Arctic over the period 2005 to 2011 (Butt et al. 2010; Muir et al. 2019). The same was seen for many PFCA: increasing concentrations were observed in ringed seals from the Canadian Arctic over the period 1992–2005(Butt et al. 2007) while more recent data show a decline from 2005 to 2010 (Muir et al. 2019), and the same was seen in ringed seals and polar bears from Greenland, where most PFAS peaked in mid 2000s and then decreased (Rigét et al. 2013).

In a time trend study of PFAS in roe deer liver from Germany 1989–2015, concentrations of PFDA, PFOA, and PFNA peaked in 2004–2006, and PFOS already in 1997 (Falk et al. 2019), a decade earlier than what is seen in Porcupine caribou in the present study as well as other biota in the Arctic. This could be expected considering roe deer in Germany probably live closer to contaminated areas; however, it peaked even before the bans of PFOS or even the voluntary cessation of production by 3M in the USA.

Nevertheless, precursors of these PFCA such as side-chain fluorinated polymers are still in many consumer products and may gradually degrade to PFCA. Thus, stable/increasing concentrations are not unexpected (Wang et al. 2015), as observed from Svalbard for arctic foxes, polar bears (Routti et al. 2017), and belugas (*Delphinapterus leucas*) (Villanger et al. 2020).

## Conclusions

The present study compared spatial and temporal PFAS concentrations in caribou/reindeer throughout the western parts of Arctic. In general, similar concentrations of all PFAS compounds were observed in Canada, Svalbard, and Isortoq, south Greenland. Caribou from Southwest Greenland (Akia and Kanger) had the highest concentrations of PFAS. Despite the remoteness, these two Greenland populations had higher PFAS concentrations than roe deer from Germany which feed at a comparable trophic level. Albeit roe deer do not utilize lichens, which caribou are adapted to digest, they are relevant for comparison because the Kanger caribou diet is primarily graminoids owing to absence of suitable lichens in that area. The higher PFAS concentrations observed in Greenland might be due to local sources nearby such as the capital (Akia caribou) as well as the main international airport and an old military base (Kanger caribou). Sea spray aerosols and lichen presence in only Akia could partly explain the differences observed between this population and Kanger caribou.

PFAS showed decreasing temporal trends during the period from early 2000s in Porcupine caribou in Canada, but not in Qamanirjuaq caribou, likely due to few sampling years. However, the same trends were not seen in Sweden, where some PFAS (PFNA, and PFHxS) increased in some populations, but were stable or decreased in the other populations. PFOS decreased in all three areas in Sweden.

The effects of PFAS on terrestrial mammals remain a large data gap in research, as reviewed by Death et al. (2021). While there are a few studies that were designed to investigate bioaccumulation and half-lives of specific PFAS in terrestrial livestock, no overt adverse effects were observed in pigs, cattle, and sheep (Death et al. 2021).

This study has demonstrated that liver concentrations of PFAS in caribou and reindeer are a useful indicator of PFAS entering terrestrial food chains from long-range transport as well as possible local sources. In addition, concentrations and patterns differ among localities, indicating different sources and human activities in different areas. Analysis of more recently collected livers from Akia and Kanger caribou are needed to elucidate the impact of potential local sources. Our study showed the importance of monitoring PFAS in Arctic caribou and reindeer over time since many areas seem to have increasing or stable concentrations despite nearby areas having decreasing concentrations. Currently, the data sets are too small to evaluate these trends in a larger perspective; hence, more analyses from recent years are required. This study has shown that continued monitoring of contaminants in caribou is important, as it provides information on background levels, but also important information for indigenous communities relying on traditional diets.

## Supplementary Information

ESM 1 (DOCX 43 kb)

ESM 2 (DOCX 19 kb)
