# Supplementary material for: Perfluoroalkyl substances in circum-ArcticRangifer: caribou and reindeer
Source: Environ Sci Pollut Res Int. 2021 Nov 23;29(16):23721–35. doi: 10.1007/s11356-021-16729-7 (PMC8979910; doi:10.1007/s11356-021-16729-7)

Supporting information, Figure S1. Time trends for PFNA, PFDA, PFUnDA, PFDoDA, PFTrDA and PFOS in Qamanirjuaq caribou, NW Canada. None of the trends are statistically significant.


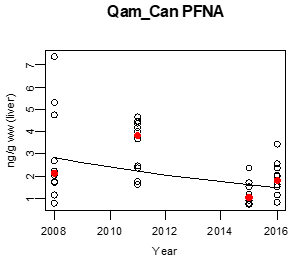

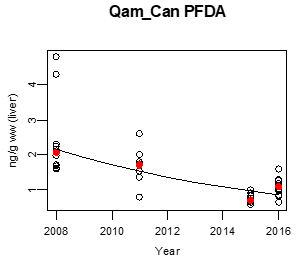

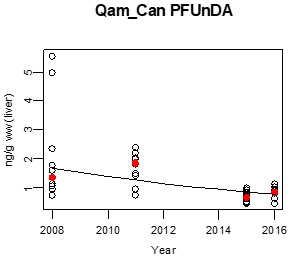

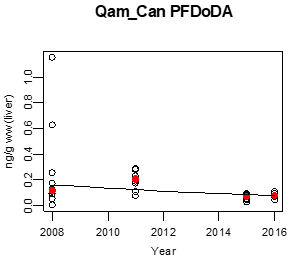

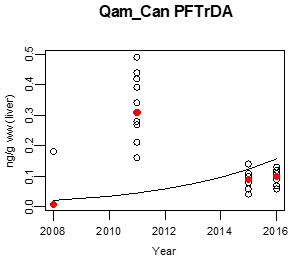

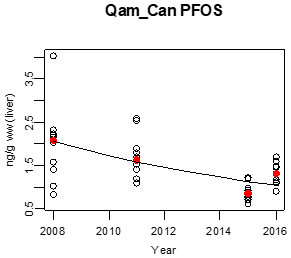

Supplement: Supplementary file 1 — (DOCX 43 kb) [file 11356_2021_16729_MOESM1_ESM.docx]
