# Supplementary material for: Perfluoroalkyl substances in circum-ArcticRangifer: caribou and reindeer
Source: Environ Sci Pollut Res Int. 2021 Nov 23;29(16):23721–35. doi: 10.1007/s11356-021-16729-7 (PMC8979910; doi:10.1007/s11356-021-16729-7)
Supplement: Supplementary file 2 — (DOCX 19 kb) [file 11356_2021_16729_MOESM2_ESM.docx]

*Supporting information Table S1. Acronym, name and method detection limits (ng/g ww) of the PFAS analyzed in the samples. na = not analyzed. nd = not detected. Numbers in bold are the lowest detected concentrations (i.e. all samples were above LOD).*

*Supporting Information, Table S2. Summary of the different analytical methods used.*

*Table S1.*

|  |  | Isortoq (South Greenland) | Canada + West Greenland | Svalbard | Sweden |
| --- | --- | --- | --- | --- | --- |
| Acronym | Name | LOD (ng/g ww) | | | |
| PFBS | Perfluorobutane sulfonic acid |  | ***0.001*** | na | 0.24 |
| PFHxS | Perfluorohexane sulfonic acid | 0.4 | 0.013 | ***0.01*** | ***0.03*** |
| PFHpS | Perfluoroheptane sulfonic acid |  | 0.013 | na | 0.01 |
| T-PFOS | Total Perfluorooctane sulfonic acid | 0.1 | 0.009 | na | na |
| L-PFOS | Branched Perfluooctane sulfonic acid |  | **0.01** | ***0.01*** | ***1.20*** |
| PFDS | Perfluorodecane sulfonic acid | 0.1 | 0.01, 0.028 | 0.1 | 0.005 |
| FOSA | Perfluorooctane sulfonic acid | 0.5 | 0.029, 0,29 | 0.5 | 0.3 |
| PFPeDA | Perfluoropentanoic acid | na | ***0.01*** | na | 0.1 |
| PFHpA | Perfluoroheptanoic acid | 0.4 | 0.016 | 0.1 | 0.176 |
| PFOA | Perfluorooctanoic acid | 0.3 | 0.014 | 0.1 | 1,314 |
| PFNA | Perfluorononoic acid | ***0.008*** | 0.008 | ***0.26*** | ***0.50*** |
| PFDA | Perfluorodecanoic acid | 0.1 | 0.008 | ***0.01*** | ***0.45*** |
| PFUnDA | Perfluoroundecanoic acid | 0.2 | 0.010 | ***0.02*** | ***0.51*** |

*Table S2.*

| Carlsson et al. 2014 | 13 C-labelled  PFBA, PFPA, PFHxA, PFHpA, PFOA, PFNA, PFDcA,  PFUnA, PFDoA, 8:2 FTCA, PFHxS, PFOS, and PFOSA | Extracted with methanol in ultrasonic bath. Centriifugation.  Cleanup on 25 mg ENVI-Carb | UHPLC tandem mass-spectrometry.  LC-MS/MS) in negative electrospray ionization | Levels in the blank were much lower (between 3 and 20 times  lower) than in the samples |
| --- | --- | --- | --- | --- |
| Herzke et al. 2009 | 13 C-labelled  PFBA, PFPA, PFHxA, PFHpA, PFOA, PFNA, PFDcA,  PFUnA, PFDoA, 8:2 FTCA, PFHxS, PFOS, and PFOSA | Extracted with  acetonitrile in an ultrasonic bath. Centriifugation. Cleanup on 25 mg ENVI-Carb | HPLC time-of-ﬂight high resolution MS in ESI negative ion mode (HPLC- ESI-ToF-MS) |  |
| Müller et al. 2011 | 13 C-labelled  PFOA, PFNA, PFDcA,  PFDoA, PFOS | Extracted with  methanol or with acetonitrile by shaking. Centriifugation. Cleanup on 25 mg ENVI-Carb | HPLC tandem mass-spectrometry.  LC-MS/MS) in negative electrospray ionization | Blank values for PFOS ranged from <0.001 – 0.15 ng/g; Median values <0.001 for all PFCAs |
| Bossi et al. 2015 | 13C PFOA, PFNA, PFDcA,  PFUnA, PFDoA, 8:2 FTCA, PFHxS, PFOS and PFOSA | tetrabutylammonium hydrogen sulphate solution+ sodium carbonate/sodium bicarbonate buﬀer and methyl-tert-butyl-ether (MTBE) by shaking. Centriifugation. | HPLC tandem mass-spectrometry.  LC-MS/MS) in negative electrospray ionization | Low levels of PFOS, PFDA, PFDoA. Much lower than in samples |
| Eriksson et al. 2016 | 13 C-labelled  PFBA, PFPA, PFHxA, PFHpA, PFOA, PFNA, PFDcA,  PFUnA, PFDoA, 13 C-6:2, 8:2, 10:2-FTUCAs, 6:2 FTSA | Extracted by skaing withacetonitrile. Cleanup on 50 mg ENVI-carb | HPLC tandem mass-spectrometry.  LC-MS/MS) in negative electrospray ionization |  |
